# Prevalence and predictors of depression among women attending antenatal care in Moshi, Tanzania: a cross-sectional study

**DOI:** 10.1186/s12884-022-04917-3

**Published:** 2022-07-26

**Authors:** James S. Ngocho, Linda M. Minja, Rimel N. Mwamba, Brandon A. Knettel, Godfrey A. Kisigo, Blandina T. Mmbaga, Melissa H. Watt

**Affiliations:** 1 Department of Epidemiology and Biostatistics, Kilimanjaro Christian Medical University College, Moshi, Tanzania; 2 Kilimanjaro Clinical Research Institute, Moshi, Tanzania; 3 Duke Global Health Institute, Duke University, Durham, NC USA; 4 School of Nursing, Duke University, Durham, NC USA; 5 Center for Global Health, Weill Cornell Medicine, New York, NY 10065 USA; 6 Mwanza Intervention Trials Unit, National Institute of Medical Research, Mwanza, Tanzania; 7 Department of Population Health Sciences, University of Utah, Salt Lake City, UT USA

**Keywords:** Tanzania, Depression, Mental health, Pregnancy, Antenatal care

## Abstract

**Introduction:**

Antenatal depression in low-and middle-income countries is under-diagnosed and leads to poorer outcomes in the pregnancy and postpartum periods. The aim of this study was to quantify depressive symptoms among pregnant women in Moshi, Tanzania, and identify factors associated with probable depression.

**Methods:**

Between March and December 2019, we enrolled 1039 pregnant women attending their first antenatal care appointment at two government health facilities to complete an audio computer-assisted self-interview. Depressive symptoms were measured with the Edinburgh Postnatal Depression Scale (EPDS), with a score > 13 indicating probable depression. A log-binomial regression model was used to identify factors associated with probable antenatal depression.

**Results:**

A total of 11.5% (119/1033) met criteria for probable depression. Depression was more common among women who were not married (16.5% vs. 7.9%, PrR = 1.5, 95% CI 1.0, 2.1) and women who reported a lifetime history of violence (22.6% vs. 5.3%, PrR = 3.3, 95% CI 2.2, 5.0). Depression was less common among women who reported more partner-specific support (PrR = 0.92, 95% CI 0.87, 0.96).

**Conclusions:**

Screening pregnant women for depressive symptoms is an essential component of evidence-based maternity care and should be accompanied by appropriate support and resources. Women who are not married, have limited support from a partner, or have experienced violence are especially vulnerable to depressive symptomatology during pregnancy.

## Introduction

Globally, an estimated 264 million people are living with depression, making it the leading mental health disorder in all age groups and a leading contributor to global burden of disease [1]. Previous research has indicated that the incidence of depression is similar in high-income and low-and middle-income countries (LMICs), with an estimated 10–20% of the population meeting criteria for depression [2, 3]. However, LMICs have much more limited availability of mental health services and mental health care professionals [4]. According to the World Health Organization (WHO), there are 278 mental health professionals serving the population of 54 million people in Tanzania, and a mere 0.06 psychiatrists for every 100,000 people, placing it among the world’s most underserved nations for mental health support [5, 6]. A lack of access to needed mental health treatment, paired with mental health stigma, is a barrier to addressing depression in this population.

Maternal mental health is an important focus due to the high prevalence of mental health challenges among pregnant and postpartum women, including antenatal depression [7, 8]. It is estimated that 15–65% of women in LMICs experience depression during pregnancy or after giving birth [7, 8]. The etiology of antenatal depression is complex and multifactorial, with factors related to hormonal changes during pregnancy and the postpartum period, the physical and emotional toll of pregnancy, childbirth and recovery, the stress of caring for an infant, and disturbances in sleep and other normal coping activities [9–11].

Antenatal depression (i.e., depression during pregnancy) is a common mood disorder among women of reproductive age [8, 12]. Risk factors associated with antenatal depression include exposure to abuse and violence, lack of social and/or partner support, and personal or family history of common mental disorders [8]. In Tanzania, higher rates of antenatal depression exist among pregnant women who have poorer relationships with their partner, and women who have a lower socio-economic status [13, 14]. In our prior study of pregnant women living with HIV in Tanzania, predictors of depression included single relationship status, food insecurity, and HIV-related shame [15]. In the Tanzanian healthcare system, there is limited attention provided to psychosocial and mental health needs during the antenatal period, which can lead to serious challenges for long-term health outcomes of the mother and child [13, 16]. With a shortage of qualified staff and irregular supply of essential equipment, drugs, and consumables, Tanzanian ANC services and clinics are often under-resourced, contributing to the disparity in addressing psychosocial and mental health needs [17].

Women who experience depression during pregnancy have poorer clinical outcomes and a variety of other health conditions, including preeclampsia, a preterm delivery, and low birth weight infants, and are more likely to have depression-related disability in the postpartum period [18]. Further, research has found that the children of mothers with depression have poorer emotional, cognitive and social development [7]. Among women living in LMICs, maternal depression has also been associated with infant risk of growth impairment, illness, and poor birth outcomes [7, 19, 20]. The impact of depression after the birth of the child has been well studied, although factors associated with depression during pregnancy have received less attention, which misses a key opportunity for targeting interventions.

The aim of this study was to identify the prevalence of probable antenatal depression and examine associated factors among pregnant women attending a first antenatal care clinic visit in Moshi, Tanzania.

## Methods

### Design and settings

This study used the baseline data from a randomized control trial of an HIV stigma reduction intervention in antenatal care (NCT 03,600,142). For this cross sectional analysis, we used baseline data from women who were enrolled at their first antenatal clinic appointment [21]. The study was conducted at two government health centers in Moshi municipality in northern Tanzania: Pasua Health Center and Majengo Health Center. These two facilities are the largest primary health care clinics in Moshi, and together they serve more than 3000 pregnant women annually.

### Population

The study included pregnant women who were at least 18 years old and attending a first ANC appointment at either of the study clinics. Women attending antenatal care were eligible for the study if they were able to understand and speak Swahili, and physically and cognitively capable of understanding and providing informed consent and completing the study survey. A total of 1047 women were enrolled at the two study clinics; of these, 8 were subsequently excluded from analysis (3 withdrew before completing the survey; 4 were later identified as ineligible; and 1 had inconsistent data). Sample size considerations for the randomized control trial have been published elsewhere [21].

### Procedures

Pregnant women attending their first antenatal care visit were identified by clinic nurses in the waiting room before participating in any clinic procedures. Women who were interested in the study were referred to a private office at the clinic to meet with research staff, learn more about the study, and continue with study procedures.

### Data collection methods

Participants completed a survey using audio computer-assisted self-interview (ACASI) technology on tablets running Questionnaire Development System (QDS) software (Nova Research Company, Version 5.0). Participants read and listened to audio recorded questions and response options. Participants selected their response using the tablet touch screen. Research assistants provided a brief tutorial of the ACASI technology, including sample questions, and were available throughout the survey to assist with participants.

The survey took approximately 40 to 60 min, and participants received 2000 Tanzanian Shillings (about 1 USD) for transportation costs.

### Variable measurement

#### Dependent variable

##### Antenatal depression

Depression was assessed using Edinburgh Postnatal Depression Scale (EPDS) [22], which has been commonly used to assess antenatal and postnatal depression in Tanzania [15, 23, 24]. The 10-item tool asks questions regarding how participants have been feeling in the past seven days, including lack of enjoyment, feelings of self-blame, sleep disturbance, unhappiness, and thoughts of harming oneself. The responses range from 0 to 3, in a four-point Likert scale, yielding a total score of 0 to 30, with higher score indicating greater symptoms (α = 0.814). Participants who scored above 13 were considered to have probable depression [25].

#### Independent variables

We collected data on demographic characteristics (e.g., age, marital status, education level), socioeconomic status (income generating activities), and health and pregnancy related variables (e.g., prior pregnancies, HIV status). In addition to the characteristics of participants, the following variables were measured. Each instrument was linguistically and culturally translated to Swahili through a team-based approach involving translation and back-translation by bilingual research personnel and open discussion to ensure cultural equivalence.

##### Perceived availability of support

General social support was assessed with the Perceived Availability of Support Scale [26]. Participants were asked questions about the support they get from people in their lives (e.g., Would someone be available to talk to you if you were upset, nervous, or depressed? Is there someone you could turn to if you needed advice to help make a decision?). This scale has a total of 8 questions with responses rated from 1 to 5, yielding a possible total score of 8 to 40, with higher score indicating more support (α = 0.866).

##### Partner support

Participants were asked four questions about the support they get from father of the child (e.g., I can count on the father of my child for financial support if I need it, The father of my child shows that he cares about me). The responses were a Likert scale of 0 to 3, with the total score ranging 0–12 and higher scores indicating more partner support (α = 0.836).

##### Lifetime Experience of Violence

Exposure to violence was assessed using 9 items in the short WHO intimate partner violence tool [27]. Women were asked to respond to how often they had experienced different types of violence (e.g., Has a spouse/partner ever insulted you or called you bad names? Has a partner/husband ever hit, slapped or hurt you physically in any other ways? As a child, were you ever forced to have some kind of sexual contact, touching, oral sex, or intercourse?). The outcome was dichotomized into two categories according to whether the participant had experienced any kind of violence (yes/no).

### Data analysis

Frequencies and percentages were used to describe categorical variables, and median and interquartile ranges were used to describe continuous variables. For scaled measures, missing data were imputed with the mean of complete items when at least 75% of items were completed. A log-binomial regression model was used to identify factors associated with probable antenatal depression. Variables that had a significance of *p* < 0.10 in univariable analysis were included in the final multivariable model. All analyses were done using Stata v16.

### Ethical consideration

Ethical approval for the study was obtained from the Kilimanjaro Christian Medical University College Research Ethics Review Committee (CRERC), the Tanzanian National Health Research Ethics Committee (NatHREC), and Duke University. All eligible participants provided informed consent before their participation; individuals who were unable to write provided a thumbprint in the presence of a witness.

In order to identify and support patients with elevated distress at risk of self-harm, we had an automatic flag in the survey to indicate if the participant answered affirmatively to the last question in the EPDS: “Thoughts of harming myself have occurred to me.” At the time of data collection, neither of the study facilities had mental health providers or services. Therefore, our study staff were trained by a clinical psychologist in the basic management and referral of these participants, using the following procedures. First, a distress form was completed, which helped to assess the severity of the thoughts and the likelihood a patient will act on them. If there were concerns about patient safety, a safety plan was completed with the patient to help the patient to identify reasons why her life was important, how to make her surroundings safer and who she could reach out to for support. Finally, if the team assessed that the participant was in immediate danger, they were advised to refer and accompany the participant to the tertiary care hospital.

## Results

The sample included 1039 participants. The median age of participants was 25 (Interquartile Range, IQR 21, 29) years. The median gestational age of the pregnancy at the time of the questionnaire was 17 weeks (IQR 12, 21). Most women (57.8%) were married. More than half (51.3%) of women had a primary school education or less, and 37.6% had no income-generating activities. More than one-third (35.8%) of women reported lifetime history of violence. A small percentage of women in the study identified as living with HIV (3.1%, as compared to local prevalence of 4.8%). More than one-third (39.4%) of participants reported that they did not know their HIV status at the time of the survey, as HIV testing is provided during the first antenatal care appointment. (Table 1).

**Table 1:** Demographic characteristics of study participants (*n* = 1039) ^a^

	**N (%)**
**Gestational age (Trimester)**	
1^st^ trimester	250 (25.2)
2^nd^ trimester	686 (69.3)
3^rd^ trimester	54 (5.4)
**Age median (IQR)**	25 (21, 29)
≤ 25 years	563 (54.2)
> 25 years	475 (45.8)
**Education**	
Primary or none	533 (51.3)
Secondary	437 (42.1)
Post-secondary	69 (6.6)
**Marital status**	
Married	599 (57.8)
Not married	438 (42.2)
**Income generating activities**	
None	390 (37.6)
Informal (e.g., selling vegetables, clothing)	491 (47.3)
Formal (i.e., salaried employment)	157 (15.1)
**First pregnancy**	
Yes	394 (38.0)
No	644 (62.0)
**HIV status**	
Negative	598 (57.6)
Positive	32 (3.1)
Unknown	409 (39.4)
**Ever experienced violence**	
Yes	371 (35.8)
No	665 (64.2)
**Presented with partner**	
Yes	548 (52.7)
No	491 (47.3)
	**Median (Q1, Q3)**
**General social support**	17 (14, 21)
**Partner support**	10 (8, 12)

^a^ Numbers may not add to total due to missing values

One hundred and nineteen (11.5%) women met the criterion for probable antenatal depression based on EPDS score. Considering the single item on suicidal ideation, 134 participants (12.9%) reported having some thoughts of self-harm in the past week (24 “quite often”, 26 “sometimes”, and 84 “hardly ever”).

Results of univariable analysis (Table 2) showed that probable depression was more prevalent among women who were not married compared with married women (PrR = 2.1, 95% CI 1.5, 3.0), women who were living with HIV compared with women who were not living with HIV or had unknown HIV status (PrR = 2.2, 95% CI 1.2, 4.1), and women who had a lifetime history of violence (PrR = 4.3, 95% CI 2.9, 6.2). Probable depression was less prevalent among women who had informal income generating activities compared to women with no generating activities (PrR = 0.7, 95% CI 0.5, 1.0), women who reported more general social support (PrR = 0.93, 95% CI 0.90, 0.95), and women who reported more partner support (PrR = 0.85, 95% CI 0.82, 0.89). Age, education level, and being pregnant for the first time were not associated with probable antenatal depression.

**Table 2:** Factors associated with probable antenatal depression (*n* = 1039) ^a^

	**Depression**		**Univariable analysis**		**Multivariable analysis**	
	**No (≤ 13)** **n(%)**	**Yes (> 13)** **n(%)**	**PrR**^**b**^** (95%CI)**	***p*-value**	**PrR (95%CI)**	***p*-value**
**Age**						
≤ 25 years	486 (87.2)	71 (12.8)	1.0			
> 25 years	428 (89.9)	48 (10.1)	0.8 (0.6 – 1.1)	0.183		
**Education**						
Primary or none	473 (89.2)	57 (10.8)	1.0			
Secondary	381 (87.8)	53 (12.2)	1.1 (0.8 – 1.6)	0.479		
High	60 (87.0)	9 (13.0)	1.2 (0.6 – 2.3)	0.565		
**Marital status**						
Married	549 (92.1)	47 (7.9)	1.0		1.0	
Not married	363 (83.5)	72 (16.5)	2.1 (1.5 – 3.0)	< 0.001	1.5 (1.0 – 2.1)	0.034
**Income activities**						
None	335 (86.1)	54 (13.9)	1.0		1.0	
Informal	441 (90.5)	46 (9.5)	0.7 (0.5 – 1.0)	0.041	0.8 (0.6 – 1.1)	0.190
Formal	138 (88.5)	18 (11.5)	0.8 (0.5 – 1.4)	0.469	0.8 (0.5 – 1.3)	0.451
**First pregnancy**						
Yes	341 (87.7)	48 (12.3)	1.1 (0.8 – 1.6)	0.521		
No	573 (89.0)	71 (11.0)	1.0			
**HIV status**						
Negative/Unknown	889 (88.9)	111 (11.1)	1.0		1.0	
Positive	25 (75.8)	8 (24.2)	2.2 (1.2 – 4.1)	0.015	1.4 (0.8 – 2.7)	0.255
**Ever experienced violence**						
Yes	287 (77.4)	84 (22.6)	4.3 (2.9 – 6.2)	< 0.001	3.3 (2.2 – 5.0)	< 0.001
No	625 (94.7)	35 (5.3)	1.0		1.0	
**General social support**	17 (14 – 21)	14 (10 – 19)	0.93 (0.90 – 0.95)	< 0.001	0.97 (0.93 – 1.00)	0.079
**Partner support**	11 (8 – 12)	8 (6 – 11)	0.85 (0.82 – 0.89)	< 0.001	0.92 (0.87 – 0.96)	< 0.001

^a^ Numbers may not add to total due to missing values

^b^ PrR = Prevalence Ratio

In the final multivariable log-binomial regression analysis, three factors (reported violence, marital status, and partner support) were associated with probable antenatal depression. Women who reported a lifetime history of violence had a higher prevalence of probable depression compared with women with no history of violence (PrR = 3.3, 95% CI 2.2, 5.0). Also, women who were not married had a higher prevalence of probable depression compared with women who were married (PrR = 1.5, 95% CI 1.0, 2.1). Women who reported more partner support had lower prevalence of probable antenatal depression (PrR = 0.92, 95% CI 0.87, 0.96). Other factors like general social support, income generating activities, and HIV status were not significantly associated with antenatal depression in the final model.

## Discussion

In this cross-sectional study, we aimed to determine the prevalence of antenatal depression, and factors associated with probable depression, among pregnant women attending their first ANC appointment in Tanzania. We found that 11.5% of women met a clinical cut-off for depression based on reported symptomatology (> 13 on the EPDS). Depression was more prevalent in women who reported a history of lifetime violence, women who were not married, and women who had low scores of partner support. Thoughts of self-harm indicative of suicide risk were also strikingly common, reported by 12.9%. With high rates of probable depression among this cohort, concentrated efforts are needed to strengthen or develop protocols for early identification of pregnant women suffering from antenatal depression and provide relevant support or treatment.

In this study, 11.5% of pregnant women attending the first ANC visit had symptoms suggestive of probable depression. Although similar findings have been previously reported in this setting [28, 29], other studies in Tanzania have reported a much higher prevalence among pregnant women. In the Tanzanian cities of Mwanza and Dar es Salaam, 33.8% and 39.5% of pregnant women screened positive for symptoms of depression, respectively [13, 30]. Several reasons might have contributed to the observed differences in the prevalence of probable depression between the studies, including study inclusion criteria, the gestational age at the time of depression screening, and the depression screening tool. Despite these differences, the fact remains that antenatal depression is of concern [31]. There is a need to screen and identify pregnant women with depression, and to intervene at early stages to avoid progression to a more advanced condition. When considering suicidal ideation, depression can be life-threatening. Similar to universal screening for HIV, universal screening for depression and mental health disorders should be part of routine antenatal care, with referral systems to link women to support and treatment.

Studies have shown that past and current events like violence and abuse (verbal or physical) play important roles in antenatal depression [32–34]. Similarly, in our study, we found that women who reported a history of violence had increased risk of depression. Childhood stressors in the form of sexual abuse are common in low- and middle-income countries, as it was shown in a multi-country study that 25% of pregnant and postpartum women had experienced childhood sexual abuse [35]. In the same study, 10 – 52% of pregnant women across multiple countries reported experiences of violence [35]. These results point to a need to screen women for a history of traumatic experiences, in order to provide support and resources. There is also a need for broader societal, legal, and policy-level advocacy to increase public awareness of violence against women, and to protect women from further abuse during pregnancy.

Consistent with previous studies, being married and having partner support were protective against antenatal depression [36]. In this survey, women who were married and women who reported having high levels of support from their partner had lower likelihood of probable depression. In Tanzania, partners are encouraged to accompany women to the first antenatal visit, and around 70% of women are accompanied by their partner [37]. The first antenatal visit presents a unique opportunity to create a strong foundation for encouraging partner support throughout the pregnancy. Partners can support pregnant women in many ways, including accompanying their partners to ANC appointments, helping them access important information, assisting in preparing the home for the new child, and providing financial and emotional support. In resource-limited settings, gender inequities often persist, and pregnant women may face major barriers in continuing their education or employment during or after a pregnancy, which leads to even greater pressure to seek support from the father of the child [33, 38, 39]. To address antenatal depression, interventions should consider opportunities to bolster partner support.

Contrary to previous studies, we found that age of the woman, HIV status, socioeconomic status at the time of pregnancy, general social support and level of education were not associated with probable depression [34, 35]. The low prevalence of HIV in this sample might have contributed to the non-significant association in our study. Also, because of challenges in measuring average household income, we had to use income-generating activity as a proxy indicator of socioeconomic status. The choice of this indicator might have influenced the magnitude of inequality, resulting in non-differential misclassification of socioeconomic status.

Strengths of this study include the robust sample size and the use of audio computer-assisted self-interview (ACASI) technology, which minimized the social desirability bias of researcher-administered surveys. Despite the strengths, the study findings need to be interpreted in light of the limitations. For example, 39.5% of women were not aware of their HIV status at enrolment. Future studies should seek to assess depression after routine HIV testing during the first ANC appointment, which would also assess the impact of a new HIV diagnosis on symptoms of depression during pregnancy. Depression was assessed using the EPDS, a screening tool, and future studies may seek to incorporate the ‘gold standard’ diagnostic interview for depression. Finally, the study excluded women under the age of 18, and therefore does not include an assessment of antenatal depression in this vulnerable population.

## Conclusion

Mental health challenges, especially depression, are common among pregnant women in low-resource settings. To improve maternal and child health outcomes, antenatal care visits should universally screen pregnant women for mental health challenges, and offer them the appropriate support, resources, and counseling.

## Data Availability

The dataset analyzed during the current study are available from the corresponding author on reasonable request.
